# Localization of SSeCKS in unmyelinated primary sensory neurons

**DOI:** 10.1186/1749-7221-3-8

**Published:** 2008-03-19

**Authors:** Christopher P Irmen, Sandra M Siegel, Patrick A Carr

**Affiliations:** 1 Dept. of Anatomy and Cell Biology, University of North Dakota, Grand Forks, ND 58202, USA

## Abstract

**Background:**

SSeCKS (*S*rc *S*uppr*E*ssed *C K*inase *S*ubstrate) is a proposed protein kinase C substrate/A kinase anchoring protein (AKAP) that has recently been characterized in the rat peripheral nervous system. It has been shown that approximately 40% of small primary sensory neurons contain SSeCKS-immunoreactivity in a population largely separate from substance P (95.2%), calcitonin gene related peptide (95.3%), or fluoride resistant acid phosphatase (55.0%) labeled cells. In the spinal cord, it was found that SSeCKS-immunoreactive axon collaterals terminate in the dorsal third of lamina II outer in a region similar to that of unmyelinated C-, or small diameter myelinated Aδ-, fibers. However, the precise characterization of the anatomical profile of the primary sensory neurons containing SSeCKS remains to be determined. Here, immunohistochemical labeling at the light and ultrastructural level is used to clarify the myelination status of SSeCKS-containing sensory neuron axons and to further clarify the morphometric, and provide insight into the functional, classification of SSeCKS-IR sensory neurons.

**Methods:**

Colocalization studies of SSeCKS with myelination markers, ultrastructural localization of SSeCKS labeling and ablation of largely unmyelinated sensory fibers by neonatal capsaicin administration were all used to establish whether SSeCKS containing sensory neurons represent a subpopulation of unmyelinated primary sensory C-fibers.

**Results:**

Double labeling studies of SSeCKS with CNPase in the dorsal horn and Pzero in the periphery showed that SSeCKS immunoreactivity was observed predominantly in association with unmyelinated primary sensory fibers. At the ultrastructural level, SSeCKS immunoreactivity was most commonly associated with axonal membrane margins of unmyelinated fibers. In capsaicin treated rats, SSeCKS immunoreactivity was essentially obliterated in the dorsal horn while in dorsal root ganglia quantitative analysis revealed a 43% reduction in the number of SSeCKS-labeled cells. This attenuation is concomitant with a decrease in fluoride-resistant acid phosphatase labeled fibers in the spinal cord dorsal horn and small neuronal somata in sensory ganglia.

**Conclusion:**

These results demonstrate that SSeCKS is primarily localized within a distinct subpopulation of small diameter, largely unmyelinated C-fiber primary sensory neurons putatively involved in nociception.

## Background

A kinase anchoring proteins (AKAPs) are a family of proteins necessary for cellular organization and compartmentalization and, as such, are likely integral components of intracellular signaling pathways [1-5]. One such AKAP, Src-suppressed C Kinase Substrate (SSeCKS), is accepted as the rodent orthologue (69% homology) of the primate specific protein gravin [6]. Gravin is expressed in an extensive array of tissue including fibroblasts, vascular endothelium, neural crest derived cells, and portions of the central (cerebellum) and peripheral (peripheral nerve, myenteric plexus and satellite cells) nervous systems [7] SSeCKS (previously identified as clone 72, [8]) has been characterized as a substrate of PKC, PKA or rho family members [9-11]. In both fibroblasts and vascular smooth muscle cells, SSeCKS has been implicated in actin-mediated cytoskeletal plasticity [12,10] that may impact cell growth, spread and adhesion [13,10,11,16]. Within the nervous system, the distribution of SSeCKS has been described [17], but the role of SSeCKS remains unresolved.

In the rodent nervous system, SSeCKS-IR has been demonstrated within rat cerebellum, the dorsal horn at all rostrocaudal spinal levels, sensory ganglia (including spinal trigeminal ganglia) and the mesencephalic nucleus of the trigeminal nerve [17]. In the dorsal root ganglia, SSeCKS-IR was localized within the cytoplasm of a specific subpopulation of small diameter neuronal perikarya. Simultaneous double labeling with classic neurochemical markers indicated that SSeCKS is found in cells that infrequently contain substance P (4.8%; SP) or calcitonin gene-related peptide (4.7%; CGRP) but more often (45%) express fluoride-resistant acid phosphatase (FRAP). In the dorsal horn of the spinal cord, double labeling for SSeCKS and acid phosphatase revealed that SSeCKS-IR fibers are localized to laminar levels dorsal to the dorsal-most third of lamina II outer. These results imply that SSeCKS may be localized within a neurochemically distinct subpopulation of C- or Aδ-fiber afferents.

In light of the uncertainty regarding the class of primary sensory neuron that contains SSeCKS, determination of the myelination status of SSeCKS-IR neurons would provide insight into the fiber classification. However, ultrastructural examination of SSeCKS containing somata or central and peripheral axonal arborizations has not been conducted. Examination of SSeCKS-IR neurons at the ultrastructural level would have implications regarding not only the myelination status, diameter and, through inference, the modality responsiveness of SSeCKS-containing primary sensory axons but also the subcellular coincidence between SSeCKS and the known distribution of PKA or components of AKAP related signaling cascade.

Capsaicin (8-methyl-N-vanillyl-6-nonenamide) is an irritant extracted from chili peppers (*Capsicum annum*) that, following systemic injection into neonatal rats, causes extensive diminution in the number of unmyelinated fibers (approximately 50% in adults) with minimal impact on the number of thinly myelinated fibers [18-21]. Functionally, the unmyelinated C-fibers that persist following neonatal capsaicin administration may represent low- and high-threshold mechanoreceptors and cold receptors [22,23]. This implies that the lesioned fibers represented chemoreceptors, heat sensitive thermoreceptors and possibly polymodal nociceptors. The effect of capsaicin is dose-dependent such that amounts much greater than 50 mg/kg almost completely obliterate unmyelinated primary sensory fibers (94%) while also eliminating small myelinated fibers by 40% [20]. At doses normally employed, neonatal capsaicin application selectively reduces the number of cells containing markers of small diameter sensory neurons such as SP, CGRP or FRAP and those cells expressing the putative capsaicin receptor, vanilloid receptor subtype-1 (VR-1). For example, the number of FRAP-containing dorsal root ganglion (DRG) neurons is decreased by approximately 65% [24,25] and the distribution of FRAP-containing terminals in the dorsal horn is almost completely attenuated. The number of primary sensory somata containing SP is reduced by approximately 90% [21] while those that contain CGRP are reduced by approximately 50–60% [26]. Not surprisingly, the number of neurons destroyed by capsaicin is similar to the number that contains the capsaicin receptor, VR-1 [27-29]. In light of the demonstrated specificity of neonatal capsaicin administration for unmyelinated primary afferents, this agent is a useful tool for the selective ablation of C-fibers.

Given that our previous study raised the possibility, but did not demonstrate, that SSeCKS primary sensory neurons are largely unmyelinated C-fibers, the neonatal capsaicin model and ultrastructural localization of SSeCKS is employed here to further investigate and clarify the functional and morphological identification of SSeCKS-IR neurons. A portion of these results has been reported in abstract form [30].

## Methods

A total of 19 Sprague-Dawley rats, of either gender, were used in this report. All procedures adhered to the appropriate animal care guidelines of the University of North Dakota and the National Institutes of Health.

### Capsaicin-induced attenuation of SSeCKS labeling

For the capsaicin study, four littermate pups (2 male; 2 female; postnatal day 5; approximately 12.5 g body weight) received an intraperitoneal injection of 50 mg/kg capsaicin (*8-methyl-N-vanillyl-nonenamide*, Sigma) in 0.9% saline with 50% ethanol. Control animals consisted of two littermates (1 male, 1 female) injected with pseudocapsaicin (postnatal day 5; 50 mg/kg;*N-vanillylnonanamide*, Sigma) in 0.9% saline with 50% ethanol and two un-injected littermates (both male). Pseudocapsaicin-injected animals served as controls for both the injection vehicle and nonspecific actions of capsaicin-like analogues. All control and capsaicin injected animals were housed together.

Animals from the capsaicin study (capsaicin-injected, pseudocapsaicin-injected, and control) were euthanized with pentobarbitol at 10 weeks of age and were perfused transcardially with 100 ml of cold (4°C) 0.9% saline containing 0.1% sodium nitrite and 0.01% heparin followed by 400 ml of cold freshly prepared fixative consisting of 4% paraformaldehyde and 0.16% para-picric acid in 0.1 M sodium phosphate buffer, pH 7.4. The spinal cord and dorsal root ganglia were removed immediately after perfusion and placed in a cold postfixative for 2 h followed by cryoprotection for at least 48 hrs in cold (4°C) 25% sucrose and 10% glycerol in 50 mM phosphate buffer.

Sensory ganglia and segmental blocks of lumbar spinal cord were fast frozen in O.C.T. embedding compound (Tissue-Tek) and cut using a cryostat at 20 μm (ganglia) or 30 μm (spinal cord) and collected into 0.1 M sodium phosphate buffer (pH 7.4) containing 0.9% saline (PBS). As described below, sections of sensory ganglia and spinal cord were then processed for SSeCKS immunolabeling alone or simultaneously double labeled for SSeCKS and VR-1. Sections of sensory ganglia and lumbar spinal cord were processed separately for FRAP enzyme histochemistry.

Sections of spinal cord or ganglia were incubated for 40–68 h at 4°C in a 1:100 dilution (in PBS containing 0.3% Triton-X; PBS-T) of anti-SSeCKS mouse monoclonal antibody (BD Transduction Labs). Labeling produced by this antibody in the rat nervous system has been previously described and characterized [17]. Following primary incubation, sections were washed twice in PBS-T, incubated 1.5 hours at room temperature with CY3-conjugated donkey anti-mouse antibody IgG (Jackson ImmunoResearch) at a dilution of 1:100 in PBS-T, then washed again in PBS-T followed by 50 mM Tris-HCl. All washes were 20 minutes. Tissue sections were mounted onto gel coated slides using 50 mM Tris HCl (pH 7.4) and coverslipped with VectaShield (Vector) anti-fade mounting medium.

Double-immunofluorescence labeling was conducted on sections from spinal ganglia and lumbar spinal cord. Sections were simultaneously incubated with SSeCKS and guinea pig polyclonal VR-1 (1:1,000; Chemicon) followed by two washes in PBS-T and incubated in CY3-conjugated donkey anti-mouse (as above) and FITC-conjugated donkey anti-guinea pig (1:100; Jackson ImmunoResearch) secondary antibodies.

FRAP enzyme histochemical procedures were conducted on sections of spinal ganglia and lumbar spinal cord as previously described [25]. Sections were first washed in 20 mM Tris-maleate (pH 5.0) for 30–60 min then reacted overnight at room temperature in a solution of filtered 6.9 mM β-glycerophosphate disodium salt hydrate, 0.9 mM lead (II) nitrate, and 0.25 μM sodium fluoride in 20 mM Tris-maleate. Sections were then visualized by a brief immersion in 2% ammonium sulfide, mounted onto glass slides from 50 mM Tris HCl (pH 7.4), and coverslipped with 9:1 glycerol: water.

Sections reacted for FRAP were analyzed using brightfield microscopy while all other immunolabeled sections were analyzed using incidental fluorescence (Olympus BX-60 or BX-50). Video images were captured using a Dage-MTI CCD-300-RC camera (at 8 bits per pixel) and Flashpoint framegrabber or a v 3.45 SPOT-RT slider digital camera (Diagnostic Instruments, Inc.). FRAP reacted sections of the lumbar spinal cord and DRG were analyzed to determine efficacy of the capsaicin treatment. Data collection for SSeCKS-IR DRG cells and for FRAP-reacted cells from both control and capsaicin-treated animals were conducted by microscopic analysis. Data from multiple animals were combined after determination that the variation and distribution were not significantly different and that the samples did not represent statistically different populations (Kolmogorov-Smirnov for normality testing and Levene median for tests of equal variance). Statistical analysis of the data from all eight animals in the capsaicin study was conducted using SigmaStat (Jandel).

### Relationship of SSeCKS with markers of myelination

Three adult Sprague-Dawley rats were perfused with 4% paraformaldehyde with picric acid fixative as described above. Sections of spinal cord, dorsal root ganglia, sciatic nerve and glabrous tissue were then processed for SSeCKS and CNPase, or Pzero double immunohistochemical labeling. Sections were simultaneously incubated with a sheep polyclonal SSeCKS (1:200; Exalpha) and either Pzero (1:200; Neuromics) or CNPase (1:200;Chemicon) primary antibodies followed by two washes in PBS-T and incubation in CY3-conjugated donkey anti-sheep (1:100; Jackson ImmunoResearch; for SSeCKS labeling) and fluorescein isothiocyanate (FITC)-conjugated donkey anti-chicken (1:100; Jackson ImmunoResearch; for Pzero labeling) or FITC-conjugated donkey anti-mouse (1:100; Jackson ImmunoResearch; for CNPase labeling) secondary antibodies. Sections were then washed in PBS-T followed by 50 mM Tris-HCl and mounted onto gel-coated slides using 50 mM Tris HCl (pH 7.4) and coverslipped with VectaShield (Vector) anti-fade mounting medium. All antibodies used in this study were well characterized and commercially available. In addition, the Exalpha SSeCKS antibody has been further characterized [17] by our lab. Omission of primary and secondary antibodies did not reveal non-specific labeling or evidence of cross-reactivity.

Analysis of labeling was conducted as described above. Analysis of colocalization (double-labeling) was conducted using photomontages of high magnification images in order to maximize resolution. All images were obtained using filter cubes optimized for CY3 (excitation filter BP 520–550; dichroic beamsplitter DM 565; bandpass barrier filter BA 580 IF) or FITC (excitation filter BP 460–490; dichroic beamsplitter DM 500; bandpass barrier filter BA 515–550 IF) in order to minimize dye cross talk or bleed-through. Confocal microscopic analysis was performed using an Olympus IX70 microscope with a Fluoview 300 PMT (Olympus) and Fluoview software (Olympus).

### Ultrastructural localization of SSeCKS

Perfusion of eight adult Sprague-Dawley rats for electron microscopy was performed as described above except with 4% paraformaldehyde/0.5% glutaraldehyde fixative. Egg imbedded tissue was sectioned (50 μm) on a Series 1000 Vibratome and incubated for 48 h at 4°C with a mouse monoclonal SSeCKS (1:100; BD Transduction Labs) antibody. Sections were washed for 40 min in PBS-T and then incubated for 1.5 with biotinylated rabbit anti-goat IgG (ABC kit, Vector) followed by avidin-biotin complex incubation for 1.5 h (ABC kit, Vector). Binding of the ABC reagent was visualized using a glucose oxidase/diaminobenzidine method (DAB; Sigma). In order to enhance labeling, glucose oxidase and nickel ammonium sulfate-intensified diaminobenzidine (GDN) were utilized. Following osmication and dehydration, the tissue was infiltrated with Durcupan and then coverslipped overnight. These sections were then mounted on Epon blocks and sectioned at ~70 nm on a RMC MTX ultramicrotome equipped with a Diatome diamond knife and placed on copper grids. Following staining with 2% uranyl acetate and lead citrate for 5 minutes each, these grids were washed and allowed to dry overnight. Prepared grids were then analyzed on a Hitachi H-7500 transmission electron microscope (TEM).

## Results

**Figure 1:**
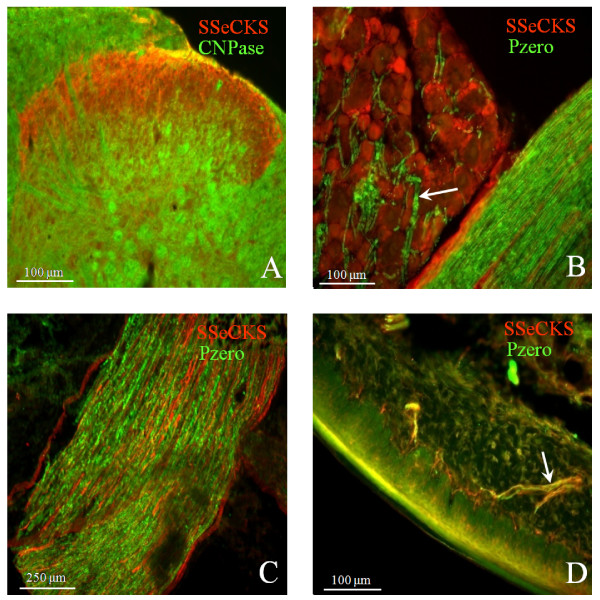
**Micrographs depicting SSeCKS colocalization with myelination markers (CNPase and Pzero).** (A) SSeCKS (red) and CNPase (green) in the lumbar spinal cord dorsal horn. The labeling appears discrete with minimal colocalization. (B) SSeCKS (red) and Pzero (green) in the L4 dorsal root ganglia. A lack of co-localization is observed and Pzero can be seen localized to putative axonal elements (arrow). (C) SSeCKS (red) and Pzero (green) in the sciatic nerve. As in the dorsal root ganglia, a lack of co-localization is observed. Both SSeCKS and Pzero can be seen localized to axonal elements. (D) SSeCKS (red) and Pzero (green) in glabrous skin of the hind-paw, fibers displaying colocalization (yellow) can be observed (arrow).

Minimal colocalization was observed between SSeCKS- and CNPase-immunoreactivity (-IR) in the lumbar dorsal horn, Lissauer's tract, and lamina X (Fig. 1A). This was confirmed using confocal microscopy, which revealed minimal colocalization between SSeCKS and CNPase except for the occasional double-labeled puncta observed in laminae ventral to the substantia gelatinosa. The CNPase labeling was primarily restricted to oligodendrocytes in deeper laminae while SSeCKS was located in Lamina I and II outer. Although both SSeCKS and CNPase labeling are found in Lissauer's tract and lamina X, double labeled fibers were not apparent.

In the dorsal root ganglion, Pzero (Schwann cell marker) labeling could be observed in association with axons that appeared to be arising from neuronal somata (Fig. 1B). However, none of the fibers with associated Pzero labeling appeared SSeCKS-immunoreactive. In peripheral nerve, colocalization of SSeCKS-labeled fibers and those associated with Pzero immunoreactivity (Fig. 1C) could not be established. However, in glabrous skin from the hindpaw, colocalization of Pzero and SSeCKS was observed toward peripheral axon terminals (Fig. 1D) and in the immediate vicinity of Meissner's corpuscles (not shown).

**Figure 2:**
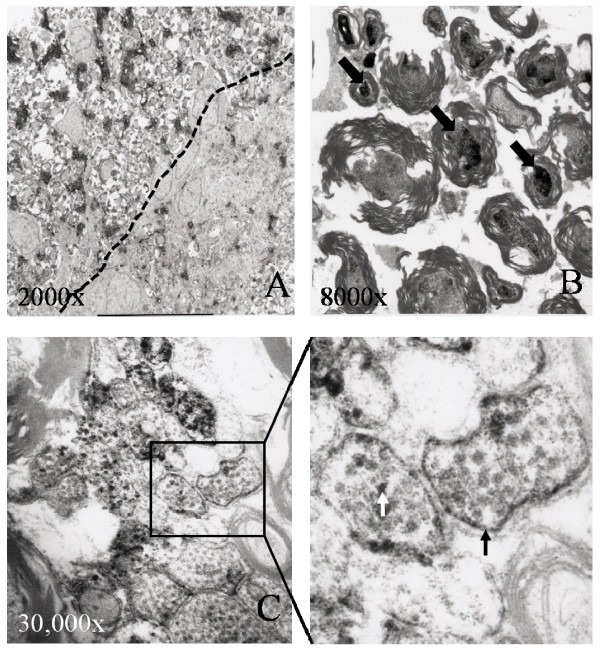
**Electron micrographs of SSeCKS-IR in the lumbar spinal cord dorsal horn.** (A) SSeCKS labeling in the dorsal horn of the spinal cord demonstrating the zone within lamina II outer in which the band of SSeCKS labeling (upper left of micrograph) is separated (dashed line) from the area of the dorsal horn lacking SSeCKS labeling (lower right of micrograph). (B) SSeCKS labeling of thinly myelinated fibers (arrows) located next to larger diameter, more heavily myelinated axons lacking SSeCKS labeling. (C) SSeCKS labeling of a bundle of unmyelinated fibers. (D) Enlargement of portion of (C) reveals labeling around the axonal plasma membrane (black arrow) of individual unmyelinated fibers, as well as labeling around vesicular structures (white arrow).

At the ultrastructural level, SSeCKS labeling was observed in the outer laminae of the lumbar dorsal horn (Fig. 2A). Specifically, SSeCKS-IR was associated with small diameter processes aggregated between larger diameter, myelinated axons. Occasionally, SSeCKS-IR could be seen within the cytoplasm of thinly myelinated axons (Fig. 2B). Comparison revealed these axons to be substantially smaller in diameter than those SSeCKS-negative axons with heavy myelination. Of the SSeCKS labeled cross-sectional profiles quantified, 15% appeared myelinated (1484 profiles counted). In those areas of the dorsal horn (ventral to lamina II) in which small diameter myelinated fibers were more abundant, myelinated SSeCKS labeled fibers were more commonly observed than non-myelinated SSeCKS labeled profiles. At the subcellular level, SSeCKS-IR was consistently localized to the plasma membrane and was frequently observed as a granular deposition throughout axonal cytoplasm (Fig. 2C, enlargement). Occasional labeling of membrane-associated vesicles was also detected. SSeCKS-IR was not found in association with any glial profiles but was found in endothelial cells as had been previously reported [12].

**Figure 3:**
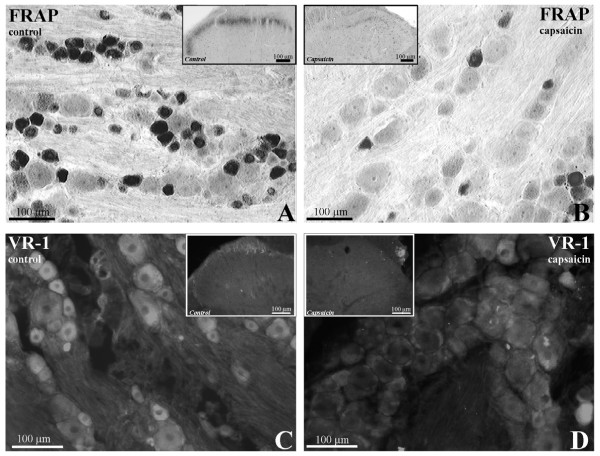
**Micrographs demonstrating capsaicin-mediated depletion of a population of primary sensory neurons.** (A) FRAP enzyme histochemical reaction product in a section of L4 DRG from a control animal. FRAP reaction product was present within approximately 40% of the small diameter cell bodies. (B) FRAP enzyme histochemical reaction product in a section of L4 DRG from a capsaicin treated animal. The number of somata containing FRAP reaction product was greatly diminished compared to untreated animals (A). (Insets A, B) FRAP enzyme histochemical reaction in transverse sections of L4 spinal cord dorsal horn from control (Inset A) and capsaicin (Inset B) animals. FRAP reaction product was localized within the dorsal third of lamina II of the dorsal horn in control animals and comparatively reduced in capsaicin treated animals. Dorsal is to top. (C) VR-1-immunofluorescence in a section of L4 DRG from a control animal. (D) VR-1-immunofluorescence in a section of L4 DRG from a capsaicin treated animal. The number of somata containing VR-1-immunofluorescence is greatly diminished compared to untreated animals (A). (Insets C, D) VR-1-immunofluorescence in transverse sections of L4 spinal cord dorsal horn from control (Inset C) and capsaicin (Inset D) animals. VR-1 staining was almost completely eliminated following capsaicin treatment.

Qualitative evaluation of the efficacy of the capsaicin administration was undertaken by comparative examination of FRAP and VR-1 labeling (Fig. 3) in sections of L4 spinal ganglia and transverse sections of L4 spinal cord from un-injected control, pseudocapsaicin-injected control and capsaicin injected animals. Pseudocapsaicin-injected and un-injected controls were comparable by all assessments. In comparison with control animals, capsaicin-injected animals demonstrated a considerable in the number of FRAP labeled small primary sensory neuron somata and central axonal collaterals (Fig. 3A, B). The robust and abundant FRAP labeling observed in small diameter primary sensory somata from control animals (Fig. 3A) was in marked contrast to the relatively limited number of small cells, either unlabeled or containing FRAP reaction product, observed in sections of DRG from capsaicin injected animals (Fig. 3B). In the dorsal horn, the intense band of FRAP reaction product in control animals (Fig. 3A inset) was almost entirely obliterated in animals treated neonatally with capsaicin (Fig. 3B inset).

As with FRAP reaction product, VR-1 labeling was substantially reduced in sections of both spinal ganglia and lumbar spinal cord (Fig. 3C,D). In sections of L4 DRG, the abundant VR-1 labeled primary sensory somata seen in control animals (Fig. 3C) were almost entirely depleted in capsaicin-injected animals (Fig. 3D). Likewise, in the dorsal horn, VR-1-immunofluorescence observed in the superficial lamina of the dorsal horn of control animals (Fig. 3C inset) was almost entirely ablated in capsaicin-injected (Fig. 3D inset) animals.

Quantitative evaluation of the efficacy of the capsaicin administration was undertaken by comparative analysis of the number of FRAP-labeled somata in control, compared to capsaicin-injected, animals. In control animals, a mean of 40.2% (± 4.4 S.D.; 7,877 cells counted from 4 animals) of DRG cells contained FRAP-enzyme reaction product whereas a mean of 25.1% (± 5.8 S.D.; 5,258 cells counted from 4 animals) DRG cells contained FRAP reactivity in animals receiving neonatal capsaicin treatment. Statistical analysis of counts was performed using a *t*-test to determine whether results from individual animals could be binned into groups. Counts from individual control animals were found to be not statistically different (*p *= 0.066) and therefore sample sets from all control animals were grouped. Likewise, counts from individual capsaicin-injected animals were also found to be statistically similar (*p *= 0.235) and therefore sample sets from all capsaicin-injected animals were grouped. Differences between the number of FRAP-labeled somata in sections of L4 DRG from control and capsaicin-injected animals were compared using an unpaired *t*-test. Capsaicin-treated animals were found to have significantly (*p *< 0.001) fewer FRAP-labeled primary sensory somata versus control animals.

**Figure 4:**
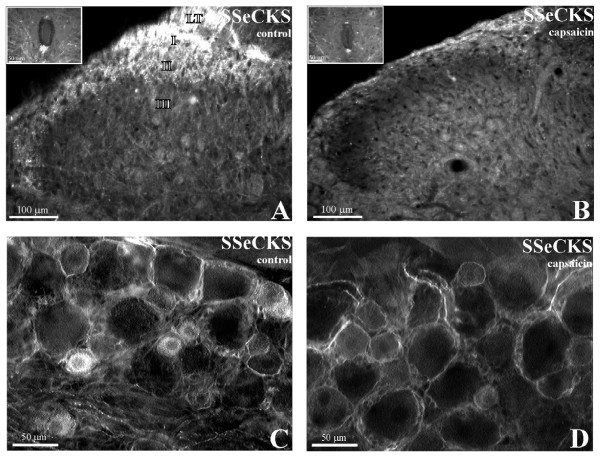
**Micrographs showing SSeCKS-immunoreacted sections of L4 DRG and spinal cord superficial dorsal horn from control and capsaicin-treated rats.** (A) In control animals, robust SSeCKS-immunofluorescence is present within Lissauer's tract (LT) and lamina I, II and dorsal lamina III. In the same section of lumbar spinal cord, brightly labeled, rostrocaudally oriented axon bundles are observed ventral to the central canal (Inset A). (B) In capsaicin-injected animals, SSeCKS-immunofluorescence within Lissauer's tract and lamina I, II and III is depleted. In the same section, the intensity and abundance of SSeCKS-labeled axons ventral to the central canal is greatly reduced (Inset B). (C) In ganglia sections from control animals, SSeCKS immunolabeling can be observed in the cytoplasm of small cells and associated with the perimeter of both small and large cells. (D) In ganglia sections from capsaicin-injected animals, a decrease in the number of small cells was quantified along with a corresponding lack of SSeCKS immunolabeling in the cytoplasm of small cells.

The effect of neonatal capsaicin administration on SSeCKS-IR in adult animals was examined in both transverse spinal cord and sensory ganglia sections. In control animals, SSeCKS-IR was similar to that previously reported [17]. In brief, SSeCKS-IR was observed in putative primary afferent central axonal arbors in Lissauer's tract, lamina I and II, as well as in occasional fibers seen to course ventrally into lamina III and IV. In addition, rostrocaudally oriented SSeCKS-IR fibers were observed both dorsal and ventral to the central canal (lamina X; Fig. 4A inset). Conversely, capsaicin-treated animals demonstrated a distinct diminution of SSeCKS-IR (Fig. 4B). The entire first and second lamina was devoid of SSeCKS-IR fibers with the rare exception of occasional faint, large diameter fibers traversing dorsoventrally through the superficial lamina. In addition, a reduction in SSeCKS-IR was observed in the area dorsal and ventral to the central canal (lamina X) of the spinal cord (Fig. 4B inset). In the dorsal columns (not shown) of capsaicin-injected animals some SSeCKS-IR persisted in apparent massed bundles of axonal processes.

In sections of spinal ganglia from control animals, SSeCKS-IR was distributed throughout the cytoplasm of sensory neuron somata with an area of increased perinuclear intensity present in most perikarya (Fig. 4C). Notwithstanding the degree of cytoplasmic labeling, intense SSeCKS-IR was often observed distributed along the somatic plasma membrane of sensory neurons of all diameters. Nuclei did not display SSeCKS-IR in any of the observed cell types. As previously reported, most cytoplasmic labeling was restricted to small diameter sensory neurons with rare large cells displaying low levels of cytoplasmically distributed SSeCKS-IR. Sections of sensory ganglia from capsaicin-treated animals revealed a considerable overall decrease in SSeCKS-IR. Most notably reduced was the number of small diameter primary sensory perikarya containing cytoplasmic SSeCKS-IR (Fig. 4D). The SSeCKS-IR that persisted in capsaicin-injected animals was not of apparent qualitative difference in localization.

Quantitative analysis from sections of L4 ganglia revealed a significant (*p *< 0.05; one-way ANOVA; Dunnett's post-hoc test) 42.5% reduction in the number of SSeCKS-labeled cells in capsaicin-treated, as compared to control, animals. In capsaicin-treated tissue, a mean of 16.9% (± 5.3 S.D.; 6,398 cells counted from 4 animals) of small cells contained SSeCKS-IR while in control tissue, a mean of 29.4% (± 5.7 S.D.; 3,903 cells counted from 4 animals) small cells were SSeCKS-IR.

## Discussion

Evaluation of colocalization between SSeCKS and CNPase at both the fluorescence and confocal microscope level suggests myelinated axons in the dorsal horn of the spinal cord contain minimal SSeCKS-IR. This finding is in agreement with our failure to find co-localization between SSeCKS and Pzero in the sciatic nerve and dorsal root ganglia. In glabrous skin, however, fluorescence and confocal microscopy revealed a colocalization of SSeCKS and Pzero in axons immediately proximal to peripheral nerve terminals. This may suggest that SSeCKS is localized in the distal-most terminations of myelinated fibers. Interestingly, Meissner's corpuscles from glabrous skin appeared innervated by axons immunoreactive for both SSeCKS and CGRP suggesting that SSeCKS may be associated with neurons that convey light touch, in addition to those conveying nociceptive signals. This observation, along with previously demonstrated innervation of Meissner's corpuscles by small, SP- or CGRP-IR fibers [31] suggests that signals from these sensory transducers may be modified by nociceptor axons.

At the ultrastructural level, the demonstration of SSeCKS labeling in bundled unmyelinated axons in dorsal horn outer laminae suggests that SSeCKS is associated with C-fibers central arborizations. However, the presence of SSeCKS-IR in occasional thinly myelinated, small diameter axons indicates that SSeCKS is not exclusively contained in C-fibers and may also be associated with a small number of A-delta fibers. The prevalence of SSeCKS-labeled unmyelinated fibers in certain areas suggests they are region-specific. In conjunction with CNPase-IR displaying minimal colocalization with SSeCKS in laminae ventral to the substantia gelatinosa, this data showing regions of SSeCKS labeled myelinated fibers in C-fiber sparse areas suggests they are located in laminae outside the substantia gelatinosa, possibly in more ventral laminae.

The subcellular localization of SSeCKS to the plasma membrane is consistent with that described for other AKAPS possessing a membrane-binding domain. This suggests that SSeCKS, like certain other AKAPs, may have a role at the axonal membrane [1,4]. This role may involve PKA-mediated intracellular signaling, possibly through adrenergic or prostaglandin receptors, similar to that reported for the human SSeCKS homologue gravin [2,3]. These ultrastructural results, in combination with the myelination status results (CNPase and Pzero) described above, strongly suggest that SSeCKS is only occasionally associated with myelinated central or peripheral primary sensory axons.

FRAP and VR-1 labeling were employed to establish the effectiveness of the neonatal capsaicin treatment. A quantitative assessment of capsaicin effectiveness, using well-established methods, was essential prior to determining its effect on SSeCKS labeling. Previous studies analyzing the effectiveness of capsaicin have traditionally used FRAP enzyme histochemistry as a marker of neuronal loss [25,32,33]. Although FRAP was used here, reports of the extensive colocalization of *Griffonia simplicifolia *isolectin I-B_4 _with FRAP [34] and LA4 with FRAP [35] suggest that SSeCKS/FRAP containing primary afferent neurons are also likely reflective of both SSeCKS/I-B_4 _and SSeCKS/LA4 subpopulations. Capsaicin treatment has been previously demonstrated to reduce the number of FRAP reactive somata in spinal ganglia by approximately 50% [25,32,33] although the degree of attenuation is dependent on many factors (e.g. dose, administration, concentration) [20]. The loss of VR1 and FRAP labeling in both DRG and spinal cord suggests that neonatal capsaicin administration was effective at eradicating a specific subpopulation of primary sensory neurons, composed of unmyelinated C-fibers and a variable degree of small-diameter myelinated (Aδ) fibers.

We previously demonstrated [17] that SSeCKS-IR is predominantly localized within small, type B somata within sensory ganglia. The large, capsaicin-induced reduction (43%) in SSeCKS labeled cells confirms that SSeCKS is at least partially localized to the unmyelinated, or small myelinated capsaicin-sensitive primary sensory neuron population. Of the SSeCKS-IR somata that persist, the pattern of intense, diffuse cytoplasmic immunoreactivity appeared similar to that observed in untreated animals.

In capsaicin treated animals, the spinal cord superficial dorsal horn, at all segmental levels, was almost completely devoid of SSeCKS immunofluorescence. The number and/or intensity of SSeCKS-IR peri-central canal fibers were also substantially diminished in capsaicin-treated animals. These findings, along with the absence of SSeCKS-IR somata within the CNS, suggest that SSeCKS-IR axons seen in the spinal cord of control animals represent the central termination of C-fiber or Aδ primary sensory neurons rather than axon terminals of descending or intrinsic spinal neurons.

Preservation of a subpopulation of SSeCKS-IR somata in the sensory ganglia with a corresponding complete loss of SSeCKS-IR fibers in the dorsal horn following neonatal capsaicin exposure suggests that those somata ablated by capsaicin represent the ganglionic component of the intensely labeled axons terminating in the dorsal horn of the spinal cord. Those SSeCKS-IR somata that persist following capsaicin treatment may contribute to the very light SSeCKS immunoreactive fibers previously reported to penetrate more ventral lamina [17].

Furthermore, the capsaicin results, taken together with the ultrastructural data revealing most SSeCKS-IR fibers to be non-myelinated, suggest that the observed SSeCKS immunoreactivity ablation was due largely to C-fiber destruction with a lesser involvement of small myelinated fibers.

## Conclusion

In light of the findings reported here, we propose that SSeCKS-IR within the peripheral nervous system is localized within a subpopulation of neurochemically distinct C-fibers and occasional thinly myelinated Aδ-fiber afferents. The myelination status and neurochemical profile of SSeCKS-IR fibers suggests that they likely convey nociceptive, thermal, or crude touch modalities and, furthermore, the presence of SSeCKS-IR fibers in the glabrous footpad is indicative of a superficial nociceptive capability. In as much as SSeCKS is an AKAP, its involvement in PKA modulated hypersensitivity is plausible and remains to be determined.

## Competing interests

The author(s) declare that they have no competing interests.

## Authors' contributions

CI performed EM analysis and myelin colocalization studies. SS performed capsaicin studies. PC was responsible for conceiving the study and revision of the final document. All authors participated in its design and coordination and helped to draft the manuscript. All authors read and approved the final document.

## Aknowledgements

This work was supported by ND EPSCoR and UNDSOMH.
